# Effect of prolactin and bromocriptine on growth of transplanted hormone-dependent mouse mammary tumours.

**DOI:** 10.1038/bjc.1977.123

**Published:** 1977-06

**Authors:** P. Briand, S. M. Thorpe, J. L. Daehnfeldt

## Abstract

Administration of ovine prolactin alone supported growth of hormone-dependent GR mouse mammary tumours. Growth of hormone-independent tumours was not stimulated. Furthermore, administration of bromocriptine, a compound that inhibits release of prolactin from the pituitary gland, was shown to inhibit the growth of hormone-dependent tumours in animals receiving treatment with progesterone + oestrone. Administration of prolactin or bromocriptine to mice bearing tumours that grew independently of progesterone + oestrone treatment had no influence on tumour growth. We conclude that direct as well as indirect evidence has been found for the involvement of prolactin in the growth of transplanted, hormone-dependent GR mouse mammary tumours.


*Br. J. Cancer* (1977) **35**, 816.

### EFFECT OF PROLACTIN AND BROMOCRIPTINE ON GROWTH OF TRANSPLANTED HORMONE-DEPENDENT MOUSE MAMMARY TUMOURS*

P. BRIAND, S. M. THORPE AND J. L. DAEHNFELDT
(with the technical assistance of M. Hansen, U. Haerslev and J. Krogsgaard Petersen)

*From The Fibiger Laboratory*†, *Ndr. Frihavnsgade* 70, *DK*-2100 *Copenhagen Ø, Denmark*

Received 28 August 1976 Accepted 25 January 1977

**Summary.—Administration of ovine prolactin alone supported growth of hormone-dependent GR mouse mammary tumours. Growth of hormone-independent tumours was not stimulated. Furthermore, administration of bromocriptine, a compound that inhibits release of prolactin from the pituitary gland, was shown to inhibit the growth of hormone-dependent tumours in animals receiving treatment with progesterone + oestrone. Administration of prolactin or bromocriptine to mice bearing tumours that grew independently of progesterone + oestrone treatment had no influence on tumour growth. We conclude that direct as well as indirect evidence has been found for the involvement of prolactin in the growth of transplanted, hormone-dependent GR mouse mammary tumours.**

CLINICAL results have demonstrated the importance of oestrogens in the growth regulation of some breast cancers (Jensen *et al.*, 1973). Other hormones, such as prolactin, may be involved in the growth of mammary cancer. Prolactin is already known to be essential for the growth of many rat mammary tumours (for review, see Boyns and Griffiths, 1972).

This communication demonstrates, directly and indirectly, that prolactin supports growth of transplantable GR mouse mammary tumours that grow in spayed mice treated with progesterone plus oestrone and do not grow in untreated spayed mice.

### MATERIALS AND METHODS

*Chemicals.*—Progesterone and oestrone were generously provided by Leo Pharmaceuticals, Ballerup, Denmark. Bromocriptine (2-Br-α-ergokryptine-methanesulphonate) was donated by Sandoz, Basel, Switzerland. Ovine prolactin (20–25 iu/mg) was supplied by Ferring AB, Malmö, Sweden. The remaining chemicals were of analytical grade.

*Tumour model.* Mammary carcinomas were induced in spayed mice of the GRS/AFib strain according to the method of van Nie (personal communication, 1970). Ten-to-12-week-old female mice were spayed and immediately treated with progesterone plus oestrone (p + o). Progesterone pellets prepared from a paste of progesterone and olive oil were injected s.c. once a week at a dose of 5–10 mg. Oestrone was dissolved in ethanol and added to the drinking water (redistilled water) at a concentration of 0·5 μg/ml.

S.c. transplantation of induced tumours was carried out using either minced tumour tissue or tumour cells isolated enzymatically according to the method of Wiepjes and Prop (1970) modified by excluding the use of DNAse. Each animal received either 100 μl of minced tumour tissue or $7–20 \times 10^6$ tumour cells.

Hormone-dependent (HD) tumours were defined as tumours that grow progressively in (p + o)-treated spayed mice but not in untreated spayed mice. Hormone-independent (HI) tumours, on the other hand, grow

* Part of this work has been presented at the Second Meeting of the European Association for Cancer Research, Heidelberg, 1973, and the Xth Acta Endocrinologica Congress, Amsterdam, 1975.

† Sponsored by the Danish Cancer Society.

equally well in (p + o)-treated and untreated spayed animals.

Ovine prolactin was dissolved in 50 mM sodium bicarbonate, pH 9·85, to yield a final concentration of 0·5 mg/ml. Aliquots were stored at −20°C. 0·5 mg prolactin was injected i.p. 3 × daily at 6–10-h intervals.

Bromocriptine was dissolved in ethanol, diluted 1/10 in $H_2O$, and 0·2 mg in 100 μl was administered s.c. daily.

*Experiments with prolactin.*—Growth of both HD and HI tumours was investigated during administration of prolactin. All the HD tumours were in the first transplant generation, while the HI tumours were either in the first or in the eighth transplant generation. In each experiment, 13–24 spayed mice received transplants of the same tumour. When the transplants had formed a tumour of 0·5–1 cm$^3$, p + o administration was discontinued in the experiments with HD tumours. Although the effects of progesterone are evident in the vaginal smear up to 4 weeks after implantation of 3 mg progesterone (Röpcke, 1975), the tumours regressed to half size within 1–2 weeks. At that time the animals were divided into 3 or 4 groups of 3–6 animals and treated as follows:

*Group I, p + o:* 5–10 mg of progesterone (3 pellets) s.c. per week + 0·5 μg/ml of oestrone in the drinking water.

*Group II, prolactin:* 50 μg (100 μl) of prolactin i.p. 3 × daily at intervals of 6–10 h.

*Group III, controls:* 100 μl of bicarbonate buffer i.p. 3 × daily at intervals of 6–10 h.

*Group IV, p + o + prolactin:* p + o treatment as in Group I, and prolactin treatment as in Group II.

Tumour growth was followed by measuring the size of the tumours with a slide caliper, as described by Rockwell, Kallman and Fajardo (1972). When the largest tumour in each group reached 1–2 cm$^3$ in size, all animals in the experiment were killed.

The experiments with HI tumours were carried out in the same way, with the modifications necessary with HI tumours. Thus, all mice received p + o treatment until the tumours had attained a volume of 0·25–0·5 cm$^3$. At this time, mice were divided into the above treatment groups (with the omission of Group IV), and tumour size was monitored as described above.

*Experiments with bromocriptine.*—Six mice were spayed for each of 5 experiments with HD tumours and immediately treated in the following manner: 2 were treated with p + o as above, 2 with p + o + bromocriptine (0·2 mg s.c. per day), and 2 untreated. One week later, tumour tissue from a single, primary tumour was transplanted to each of the 6 animals. HI tumours were transplanted to 4 animals only, 2 receiving treatment with bromocriptine and 2 untreated.

The animals were observed twice weekly. When the largest tumour reached approximately 1 cm$^3$ in size, all animals within the single experiment were killed, and the tumours were removed and weighed.

*Statistics*

Statistical evaluation was performed with the non-parametric Wilcoxon rank-sum test.

### RESULTS

The effects of prolactin, p + o + prolactin, and p + o, on regrowth of regressing HD mammary tumours, were studied in 6 experiments. The results are shown in Fig. 1. In all 6 experiments, treatment with prolactin alone supported tumour growth for several days, whereas tumours continued to regress in all control mice injected only with buffer. In each of the experiments, the final volume of the tumours in the prolactin-treated group was significantly higher ($P \leqslant 0·01$) than that of the tumours in the control groups. Futhermore, prolactin significantly stimulated ($P \leqslant 0·02$) (p + o)-supported tumour growth in 1 (Experiment D) of 3 experiments. Administration of prolactin had no significant effect ($P >$ 0·10) on the growth of HI tumours (Fig. 2).

The growth of HD tumours transplanted to spayed mice receiving bromocriptine + p + o was studied in 5 experiments. In all of these, mean tumour volume was lower in animals treated with bromocriptine + p + o than in controls treated with p + o alone (Fig. 3). This difference is statistically significant ($P < 0·05$) as tested by the

FIG. 1.—Effect of administration of prolactin and/or p + o on growth of HD mammary carcinomas in spayed GR mice. All tumours were in the first transplant generation. Administration of p + o to spayed mice bearing HD tumours was discontinued when tumours reached 0·5–1 $cm^3$. Subsequently, tumours began to regress. 1–2 weeks later (Day 0), the animals were divided into the following groups for treatment with various hormones. I: 5–10 mg of progesterone (p) pellets s.c. weekly + 0·5 μg/ml of oestrone (o) in the drinking water. II: 50 μg (100 μl) of prolactin i.p.3 × daily at intervals of 6–10 h. III: 100 μl of buffer i.p. 3 × daily at intervals of 6–10 h. IV: p + o as in I + prolactin as in II. Relative tumour volume is defined as

$$\frac{\text{Tumour volume at a given time}}{\text{Tumour volume at Day 0}}$$

Each point on the curves represents the median of 5–6 tumours, except Expt. B, Group I, in which only 3 mice are included. The range of relative tumour volume at the end of each experiment is shown by bars.

FIG. 2.—Effect of administration of prolactin or p + o on growth of HI mammary carcinomas in spayed GR mice. Tumours of Expt. G were in the 1st transplant generation, and tumours of Expt. H in the 8th transplant generation. When HI tumour transplants had attained a volume of 0·25–0·5 $cm^3$ (Day 0), the mice were divided into the following groups for treatment with hormones: I: 5–10 mg of progesterone (p) pellets s.c. weekly + 0·5 μg/ml of oestrone (o) in the drinking water. II: 50 μg (100 μl) of prolactin i.p. 3 × daily at intervals of 6–10 h. III: 100 μl of buffer i.p. 3 × daily at intervals of 6–10 h. Relative tumour volume is defined as

$$\frac{\text{Tumour volume at a given time}}{\text{Tumour volume at Day 0}}$$

Each point on the curves represents the median of 5–6 tumours. The range of relative tumour volume at the end of each experiment is shown by bars.

Wilcoxon rank-sum test based on all single observations shown in the figure. Thus the zero value in Experiment 5 is also included. No tumour takes were observed in the untreated mice. In contrast, when HI tumours were transplanted to mice receiving bromocriptine, tumour growth in treated mice did not differ from that in the untreated, control mice (Fig. 4). In Experiments 9b and 10b, bromocriptine seemed to inhibit tumour growth, but in duplicate experiments with the same tumour line, this did not appear to be the case (Experiments 9a and 10a).

FIG. 3.—Growth of HD mammary carcinomas in spayed GR mice treated with p + o (open circles) or p + o + bromocriptine (closed circles). All tumours were in the 1st transplant generation. In each experiment, one primary tumour was transplanted to 6 spayed mice, 2 receiving 5–10 mg of progesterone (p) pellets s.c. weekly + 0·5 μg/ml of oestrone (o) in the drinking water, 2 receiving p + o + daily injections of 0·2 mg bromocriptine s.c., and 2 untreated. The number of days that tumour-bearing mice received treatment is shown below the Fig. The open and closed circles denote mean tumour weight of 2 tumours, the individual weights of which are indicated by the ends of the bars. No tumours were observed in untreated spayed mice at the end of the experiment (not shown in Fig.).

FIG. 4.—Growth of HI mammary carcinomas in spayed GR mice, untreated (open circles) or treated with bromocriptine (closed circles). In Expts 7–8, 1 tumour, and in Expts 9–11, 2 tumours (a and b), were each transplanted to 4 spayed mice, 2 untreated and 2 given daily injections of 0·2 mg bromocriptine s.c. The number of days that tumour-bearing mice received treatment is shown below the Fig. The open and closed circles denote mean tumour weight of 2 tumours, the individual weights of which are indicated by the ends of the bars.

### DISCUSSION

The importance of prolactin in mammary tumour induction has previously been established in rats and mice by demonstrating a significantly higher tumour incidence under various conditions leading to increased serum prolactin levels (Boot, 1970; Meites, 1972), and by demonstrating a significantly lower tumour incidence during administration of bromocriptine, an inhibitor of prolactin secretion (Stähelin, Burckhardt-Vischer and Flückiger, 1972; Yanai and Nagasawa, 1972; Welsch and Gribler, 1973; Chan and Cohen, 1974).

Prolactin has also been shown to stimulate the growth of pre-existing mammary tumours in DMBA-treated rats (Pearson *et al.*, 1969; Welsch, Clemens and Meites, 1969; Nagasawa and Yanai, 1970; Leung and Sasaki, 1975). Furthermore, when prolactin secretion is diminished in carcinogen-treated rats (by administration of bromocriptine), mammary tumour growth has been found to be inhibited (Heuson, Waelbroeck-Van Gaver and Legros 1970; Nagasawa and Meites, 1970).

In the mouse, the role of prolactin in mammary tumour growth has been less thoroughly investigated. Most mouse mammary tumours are HI (Mühlbock, 1955). However, in a few inbred strains, such as the BR strains (Foulds, 1949), the DD strain (Heston, Vlahakis and Tsubura,

1964), the RIII strain (Squartini, 1962), and the GR strain (van Nie and Thung, 1965), the incidence of pregnancy-responsive mammary tumours is very high. Studies of the effects of pregnancy and pseudo-pregnancy on mammary tumour induction and growth in BR6 mice indicated that the hormones of late pregnancy were important for the induction of new tumours, whereas the hormones of early pregnancy stimulated the growth of existing tumours (Lee, 1970). Since prolactin levels are high in early pregnancy in the mouse (Murr, Bradford and Geschwind, 1974), it is feasible that prolactin is involved in the growth of these pregnancy-dependent tumours. The finding in BR6 mice that 4/7 tumours induced by grafting pituitary glands under the kidney capsule ceased to grow after removal of the graft-bearing kidney, offers further indication for the involvement of prolactin in the growth of mouse mammary tumours.

In the GR strain of mice, mammary tumours can be induced by forced breeding (van Nie and Dux, 1971) or by treatment of spayed mice with p + o (van Nie, personal communication,). Most of the mammary tumours induced by p + o treatment are dependent upon the continued administration of p + o for growth (Briand and Daehnfeldt, 1973; Sluyser and van Nie, 1974). Since long-sustained administration of oestrogens is known to lead to enlargement of the pituitary gland and increased production of prolactin in mice (Kim, Furth and Yannopoulos, 1963; Boot, Kwa and Röpcke, 1973; Chan and Cohen, 1974) prolactin might be involved in the stimulation of growth of these (p + o)-dependent GR mouse mammary tumours.

We have found that administration of prolactin alone leads to regrowth of regressing GR mouse mammary tumours. The growth rate of tumours in mice treated with prolactin was not, however, as great as that in the (p + o)-treated mice. This may have been due to use of heterologous prolactin or maintenance of suboptimal levels of prolactin throughout part of the experiment. Provided that the half-life of prolactin in mice is of the same order as that found in rats (Kwa *et al.*, 1970), very low prolactin levels would have prevailed, in spite of the fact that a very high dose of prolactin was administered. Yet another possible explanation for the above observation would be that prolactin may require oestrogen to exert its maximal effect on tumour growth, or *vice versa*. However, while the effects of p + o + prolactin on tumour were additive in 1/3 experiments (Fig. 1, experiment D), a potentiation of the hormone effects was not observed.

(p + o)-dependent growth was inhibited by bromocriptine. In 4/5 experiments (bromocriptine + p + o)-treated animals showed lower values than (p + o)-treated controls, without any overlapping (see Fig. 3). In the fifth experiment one of the control mice for unknown reasons did not develop any tumour. The other one produced a tumour which weighed more than the tumours in the 2 mice which received bromocriptine.

When the results of all 5 experiments were evaluated by the Wilcoxon rank-sum test the inhibitory effect of bromocriptine was found to be statistically significant. An unspecific, toxic effect of bromocriptine on tumour growth is unlikely but cannot be excluded, since animals bearing HD tumours were treated with bromocriptine for a longer period of time than animals bearing HI tumours.

A difference in time of treatment was unavoidable, since the same amount of tumour tissue was transplanted to each mouse, and the treatment period was not extended beyond the exponential growth phase of the tumour.

In conclusion, evidence has been presented for the involvement of prolactin in the growth of transplanted, hormone-dependent GR mouse mammary tumours.

We want to thank K. Roholt, Leo Pharmaceuticals, Ballerup. Denmark, for the generous supply of oestrone and progesterone. We are grateful to E. Flückiger, Sandoz, Basel, Switzerland, for supplying us with bromocriptine, and to Ferring, Malmö, for providing us with ovine prolactin.

This work was supported by the Danish Cancer Society and a grant from Mrs Agathe Neye. S. M. Thorpe was supported by a scholarship from the Medical Faculty of the University of Copenhagen.

### REFERENCES

Boot, L. M. (1970) Prolactin and Mammary Gland Carcinogenesis. The Problem of Human Prolactin. *Int. J. Cancer*, **5**, 167.

Boot, L. M., Kwa, H. G. & Röpcke, G. (1973) Radioimmunoassay of Mouse Prolactin. Prolactin Levels in Isograft-bearing Orchidectomized Mice. *Eur. J. Cancer*, **9**, 185.

Boyns, A. R. & Griffiths, K. (1972) *Prolactin and Carcinogenesis*. Cardiff: Alpha Omega Alpha.

Briand, P. & Daehnfeldt, J. L. (1973) Enzyme Patterns of Glucose Catabolism in Hormone-dependent and -independent Mammary Tumours of GR Mice. *Eur. J. Cancer*, **9**, 763.

Chan, P.-C. & Cohen, L. A. (1974) Effects of Dietary Fat, Antiestrogen, and Antiprolactin on the Development of Mammary Tumors in Rats. *J. nqtn. Cancer Inst.*, **52**, 25.

Foulds, L. (1949) Mammary Tumours in Hybrid Mice: Growth and Progression of Spontaneous Tumours. *Br. J. Cancer*, **3**, 345.

Heston, W. E., Vlahakis, G. & Tsubura, Y. (1964) Strain DD, a new High Mammary Tumor Strain, and Comparison of DD with Strain C3H. *J. natn. Cancer Inst.*, **32**, 237.

Heuson, J. C., Waelbroeck-Van Gaver, C. & Legros, N. (1970) Growth Inhibition of Rat Mammary Carcinoma and Endocrine Changes Produced by 2-Br-α-Ergocryptine, a Suppressor of Lactation and Nidation. *Eur. J. Cancer*, **6**, 353.

Jensen, E. V., Block, G. E., Smith, S. & Desombre, E. R. (1973) Hormonal Dependency of Breast Cancer. *Recent Results Cancer Res.*, **42**, 55. Ed. M. L. Griem, E. W. Jensen, S. E. Ultmann & R. W. Wissler. Berlin: Springer-Verlag.

Kim, U., Furth, J. & Yannopoulos, K. (1963) Observatons on Hormonal Control of Mammary Cancer. I. Estrogen and Mammatropes. *J. natn. Cancer Inst.*, **31**, 233.

Kwa, H. G., Feltkamp, C. A., van der Gugten, A. A. & Verhofstad, F. (1970) The Rate of Elimination of Prolactin as a Determinant Factor for Plasma Levels Assayed in Rat. *J. Endocr.*, **48**, 299.

Lee, A. E. (1970) Mammary Tumour Development in BR6 Mice: Ovarian Influences and 5-Hydroxytryptamine. *Br. J. Cancer*, **24**, 561.

Leung, B. S. & Sasaki, G. H. (1975) On the Mechanism of Prolactin and Estrogen Action in 7,12-Dimethylbenz(a)anthracene-induced Mammary Carcinoma in the Rat. II. *In vivo* Tumor Responses and Estrogen Receptor. *Endocrinology*, **97**, 564.

Meites, J. (1972) Relation of Prolactin to Mammary Tumourigenesis and Growth in Rats. In *Prolactin and Carcinogenesis*. Ed. A. R. Boyns & K. Griffiths. Cardiff: Alpha Omega Alpha.

Murr, S. M., Bradford, G. E. & Geschwind, I. I. (1974) Plasma Luteinizing Hormone, Follicle-stimulating Hormone and Prolactin during Pregnancy in the Mouse. *Endocrinology*, **94**, 112.

Mühlbock, O. (1955) The Influence of Hormones on Spontaneous Experimental Tumours. In *Simposio sugli antimitotica* (San Remo), p. 41.

Nagasawa, H. & Meites, J. (1970) Suppression by Ergocornine and Iproniazid of Carcinogen-induced Mammary Tumors in Rats: Effects on Serum and Pituitary Prolactin Levels. *Proc. Soc. exp. Biol. Med.*, **135**, 469.

Nagasawa, H. & Yanai, R. (1970) Effects of Prolactin or Growth Hormone on Growth of Carcinogen-induced Mammary Tumours of Adreno-ovariectomized Rats. *Int. J. Cancer*, **6**, 488.

Pearson, O. H., Llerena, O., Llerena, L., Molina, A. & Butler, T. (1969) Prolactin-dependent Rat Mammary Cancer: A Model for Man? *Trans. Assoc. Am. Physicians*, **82**, 225.

Rockwell, S. C., Kallman, R. F. & Fajardo, L. F. (1972) Characteristics of a Serially Transplanted Mouse Mammary Tumor and its Tissue-culture-adapted Derivative. *J. natn. Cancer Inst.*, **49**, 735.

Röpcke, G. (1975) *Interaction of Hypophyseal Isografts and Ovarian Hormones in Mammary Tumour Development in Mice.* Thesis. p. 57. Amsterdam: Mondeel.

Sluyser, M. & van Nie, R. (1974) Estrogen Receptor Content and Hormone-responsive growth of Mouse Mammary Tumors. *Cancer Res.*, **34**, 3253.

Squartini, F. (1962) Responsiveness and Progression of Mammary Tumors in High-cancer-strain Mice. *J. natn. Cancer Inst.*, **28**, 911.

Stähelin, H., Burckhardt-Vischer, B. & Flückiger, E. (1971) Rat Mammary Cancer Inhibition by a Prolactin Suppressor, 2-Bromo-α-ergokryptine (CB154). *Experientia*, **27**, 915.

van Nie, R. & Dux, A. (1971) Biological and Morphological Characteristics of Mammary Tumors in GR Mice. *J. natn. Cancer Inst.*, **46**, 885.

van Nie, R. & Thung, P. J. (1965) Responsiveness of Mouse Mammary Tumours to Pregnancy. *Eur. J. Cancer*, **1**, 41.

Welsch, C. W., Clemens, J. A. & Meites, J. (1969) Effects of Hypothalamic and Amygdaloid Lesions on Development and Growth of Carcinogen-induced Mammary Tumors in the Female Rat. *Cancer Res.*, **29**, 1541.

Welsch, C. W. & Gribler, C. (1973) Prophylaxis of SpontaneouslyDeveloping Mammary Carcinoma in C3H/HeJ Female Mice by Suppression of Prolactin. *Cancer Res.*, **33**, 2939.

Wiepjes, G. J. & Prop, F. J. A. (1970) Improved Method for Preparation of Single-cell Suspensions from Mammary glands of Adult Virgin Mouse. *Expl Cell Res.*, **61**, 451.

Yanai, R. & Nagasawa, H. (1972) Inhibition of Mammary Tumorigenesis by Ergot Alkaloids and Promotion of Mammary Tumorigenesis by Pituitary Isografts in Adreno-ovariectomized Mice. *J. natn. Cancer Inst.*, **48**, 715.


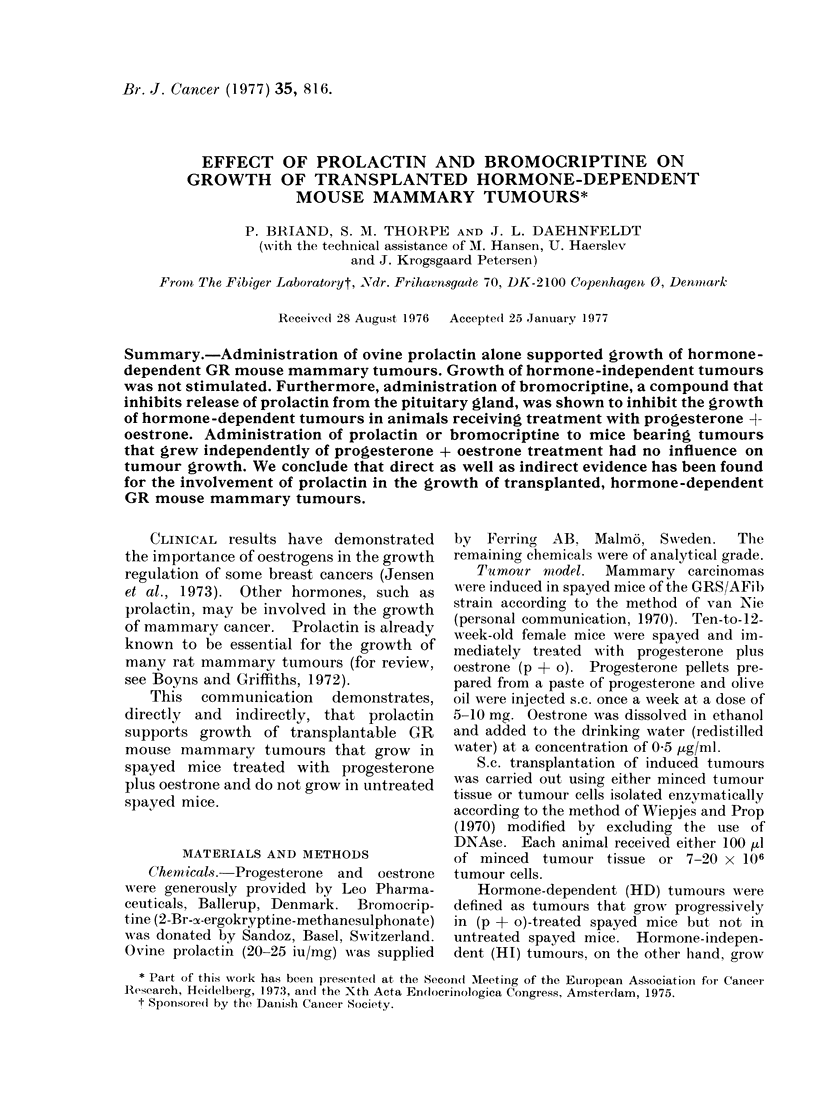

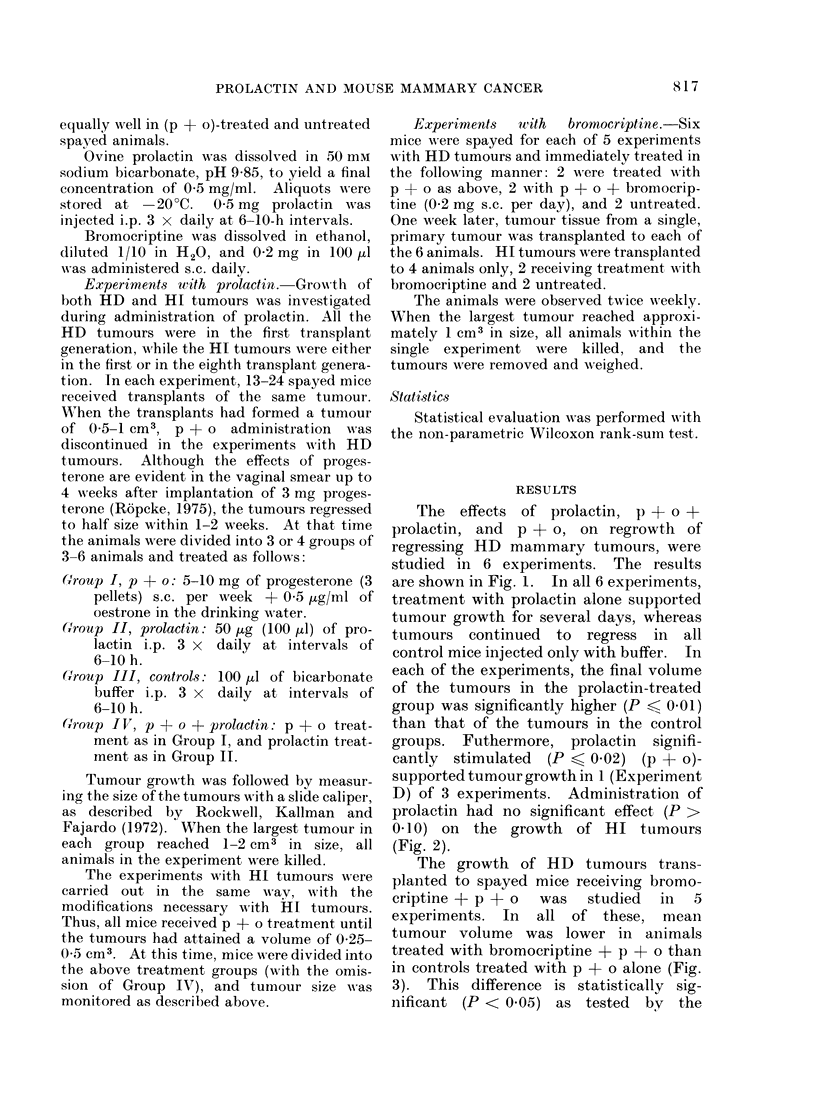

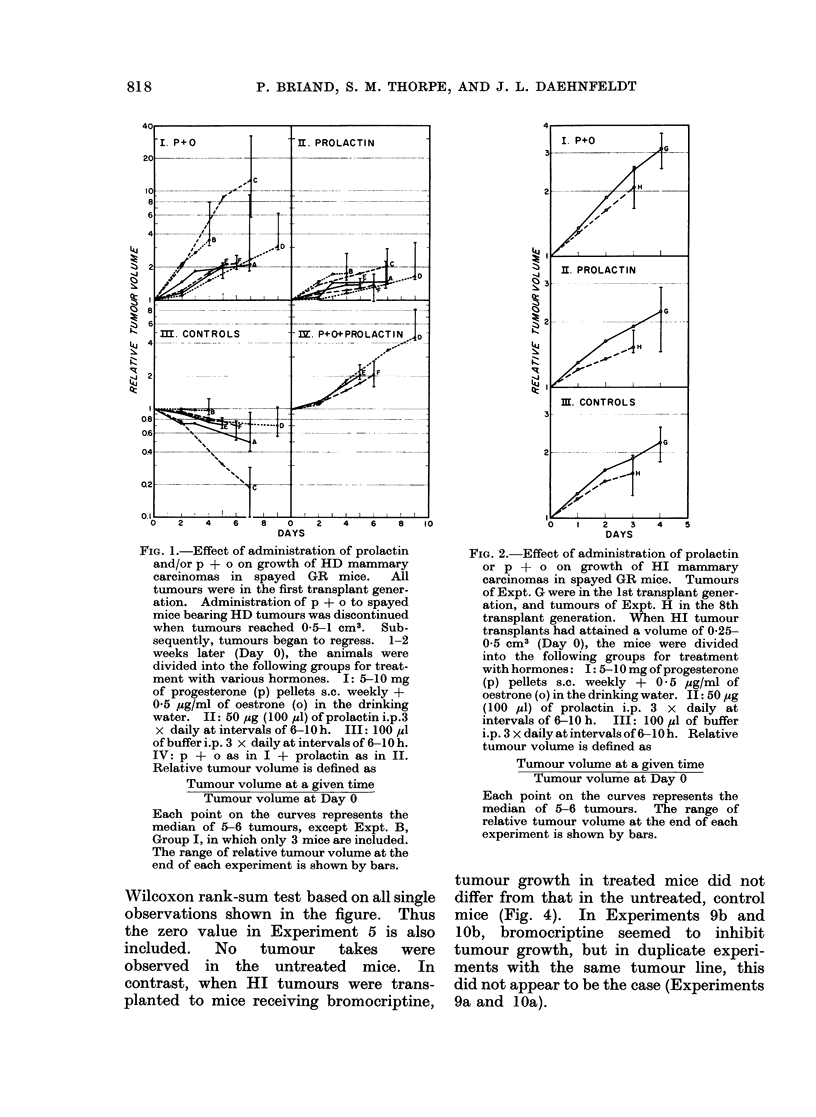

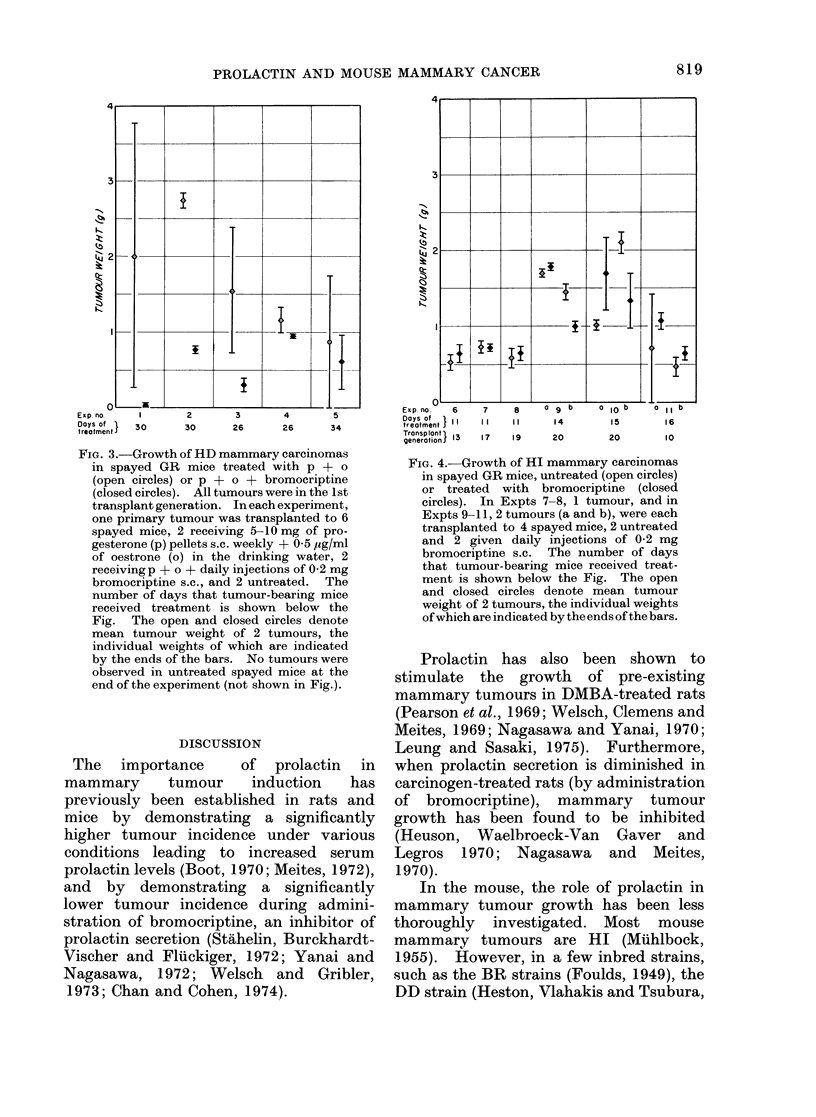

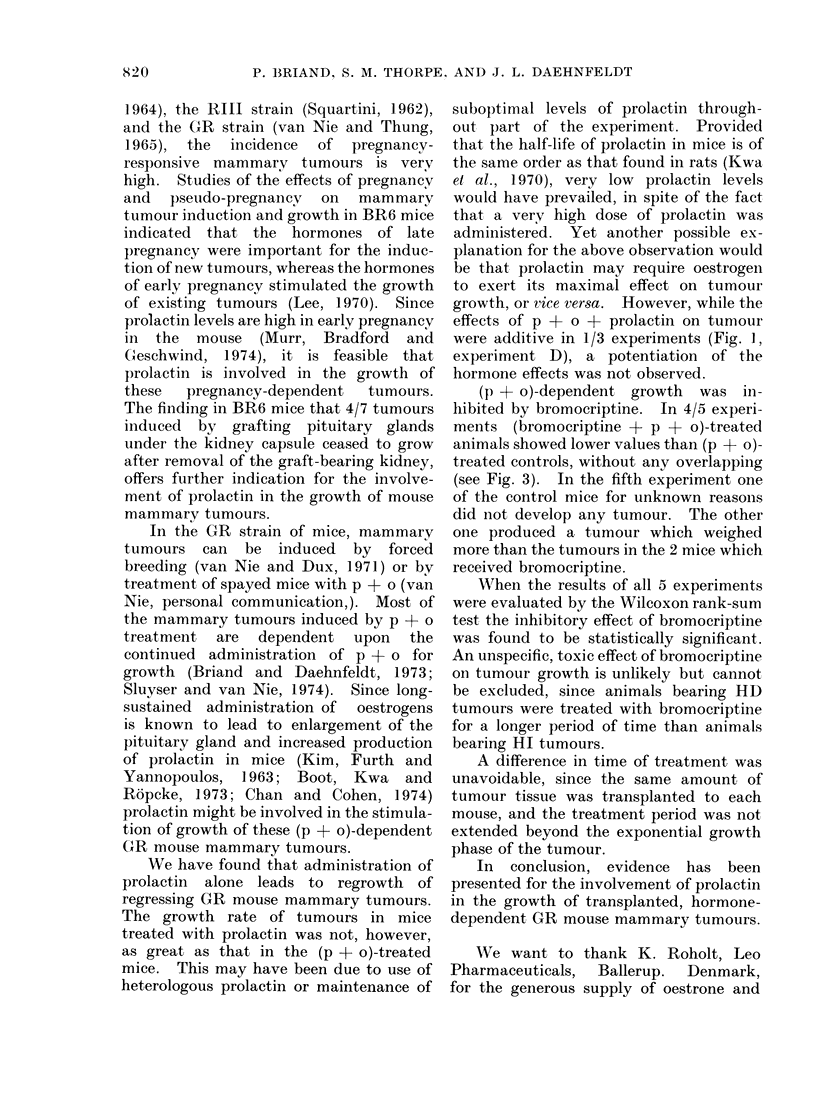

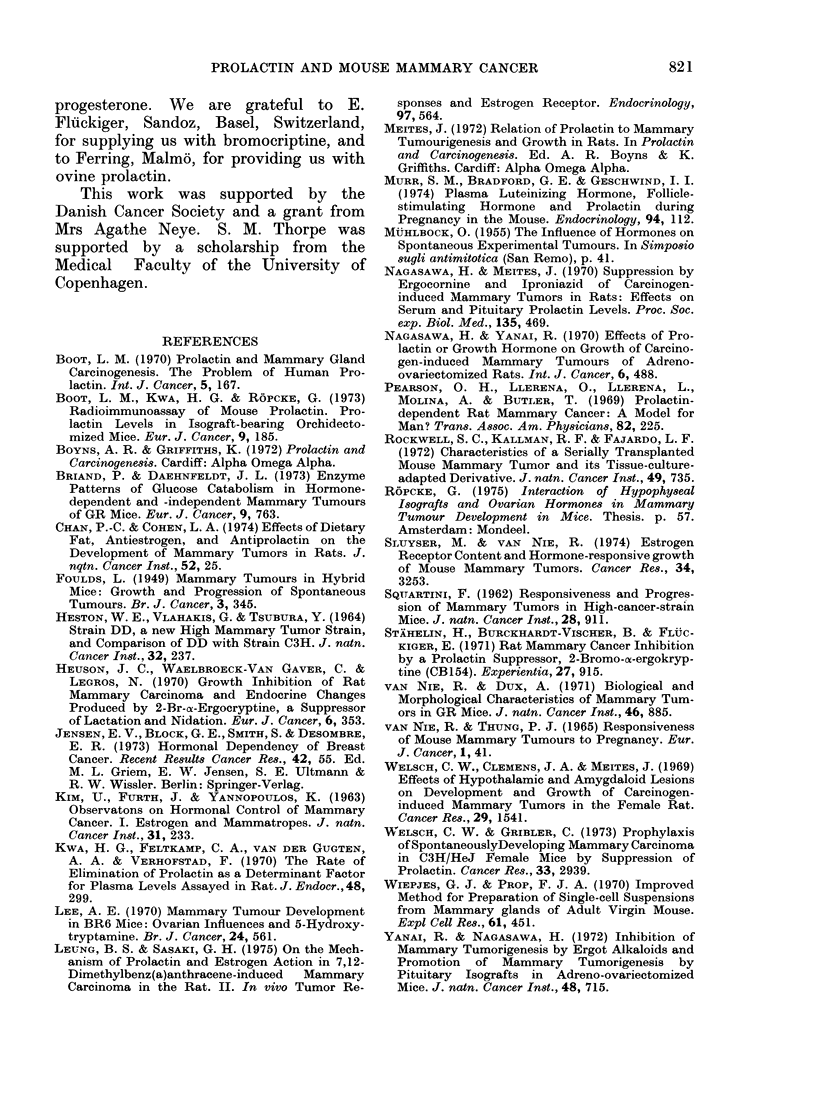


## References

[OCR_00559] Boot L. M., Kwa H. G., Röpcke G. (1973). Radioimmunoassay of mouse prolactin. Prolactin levels in isograft-bearing orchidectomized mice.. Eur J Cancer.

[OCR_00554] Boot L. M. (1970). Prolactin and mammary gland carcinogenesis. The problem of human prolactin.. Int J Cancer.

[OCR_00569] Briand P., Daehnfeldt J. L. (1973). Enzyme patterns of glucose catabolism in hormone-dependent and -independent mammary tumours of GR mice.. Eur J Cancer.

[OCR_00575] Chan P. C., Cohen L. A. (1974). Effect of dietary fat, antiestrogen, and antiprolactin on the development of mammary tumors in rats.. J Natl Cancer Inst.

[OCR_00581] FOULDS L. (1949). Mammary tumours in hybrid mice; growth and progression of spontaneous tumours.. Br J Cancer.

[OCR_00586] HESTON W. E., VLAHAKIS G., TSUBURA Y. (1964). STRAIN DD, A NEW HIGH MAMMARY TUMOR STRAIN, AND COMPARISON OF DD WITH STRAIN C3H.. J Natl Cancer Inst.

[OCR_00592] Heuson J. C., Waelbroeck-van Gaver C., Legros N. (1970). Growth inhibition of rat mammary carcinoma and endocrine changes produced by 2-Br-alpha-ergocryptine, a suppressor of lactation and nidation.. Eur J Cancer.

[OCR_00605] KIM U., FURTH J., YANNOPOULOS K. (1963). OBSERVATIONS ON HORMONAL CONTROL OF MAMMARY CANCER. I. ESTROGEN AND MAMMOTROPES.. J Natl Cancer Inst.

[OCR_00611] Kwa H. G., Feltkamp C. A., Verhofstad F., van der Gugten A. A. (1970). The rate of elimination of prolactin as a determinant factor for plasma levels assayed in rats.. J Endocrinol.

[OCR_00618] Lee A. E. (1970). Mammary tumour development in BR6 mice: ovarian influences and 5-hydroxytryptamine.. Br J Cancer.

[OCR_00623] Leung B. S., Sasaki G. H. (1975). On the mechanism of prolactin and estrogen action in 7,12 dimethylbenz(A)anthracene-induced mammary carcinoma in the rat. II. In vivo tumor responses and estrogen receptor.. Endocrinology.

[OCR_00638] Murr S. M., Bradford G. E., Geschwind I. I. (1974). Plasma luteinizing hormone, follicle-stimulating hormone and prolactin during pregnancy in the mouse.. Endocrinology.

[OCR_00648] Nagasawa H., Meites J. (1970). Suppression by ergocornine and iproniazid of carcinogen-induced mammary tumors in rats; effects on serum and pituitary prolactin levels.. Proc Soc Exp Biol Med.

[OCR_00655] Nagasawa H., Yanai R. (1970). Effects of prolactin or growth hormone on growth of carcinogen-induced mammary tumors of adreno-ovariectomized rats.. Int J Cancer.

[OCR_00661] Pearson O. H., Llerena O., Llerena L., Molina A., Butler T. (1969). Prolactin-dependent rat mammary cancer: a model for man?. Trans Assoc Am Physicians.

[OCR_00667] Rockwell S. C., Kallman R. F., Fajardo L. F. (1972). Characteristics of a serially transplanted mouse mammary tumor and its tissue-culture-adapted derivative.. J Natl Cancer Inst.

[OCR_00678] Sluyser M., Van Nie R. (1974). Estrogen receptor content and hormone-responsive growth of mouse mammary tumors.. Cancer Res.

[OCR_00705] Welsch C. W., Clemens J. A., Meites J. (1969). Effects of hypothalamic and amygdaloid lesions on development and growth of carcinogen-induced mammary tumors in the female rat.. Cancer Res.

[OCR_00712] Welsch C. W., Gribler C. (1973). Prophylaxis of spontaneously developing mammary carcinoma in C3H-HeJ female mice by suppression of prolactin.. Cancer Res.

[OCR_00718] Wiepjes G. J., Prop F. J. (1970). Improved method for preparation of single-cell suspensions from mammary glands of adult virgin mouse.. Exp Cell Res.

[OCR_00724] Yanai R., Nagasawa H. (1972). Inhibition of mammary tumorigenesis by ergot alkaloids and promotion of mammary tumorigenesis by pituitary isografts in adreno-ovariectomized mice.. J Natl Cancer Inst.

[OCR_00695] van Nie R., Dux A. (1971). Biological and morphological characteristics of mammary tumors in GR mice.. J Natl Cancer Inst.

[OCR_00700] van Nie R., Thung P. J. (1965). Responsiveness of mouse mammary tumours to pregnancy.. Eur J Cancer.

